# Zinc supplementation and COVID-19 mortality: a meta-analysis

**DOI:** 10.1186/s40001-022-00694-z

**Published:** 2022-05-23

**Authors:** Seyed-Amir Tabatabaeizadeh

**Affiliations:** Department of Nutrition Sciences, Varastegan Institute for Medical Sciences, Mashhad, Iran

**Keywords:** SARS-CoV-2, COVID-19, Zinc, Mortality, Meta-analysis

## Abstract

**Background and aims:**

Severe acute respiratory syndrome coronavirus 2 (SARS-CoV-2) is the agent of a pneumonia outbreak and was called 2019 novel coronavirus disease (COVID-19). COVID-19 emerged in December 2019 and now considered a pandemic. Zinc supplementation can reduce mortality in patients with severe pneumonia. This study aimed at meta-analysis of the results of related studies and evaluate the effect of zinc supplementation on COVID-19 mortality.

**Methods:**

A systematic search has conducted for manuscripts through PUBMED/Medline and Google Scholar (Cochrane guideline has considered it as the gray literature) up to September 2021. This meta-analysis followed Preferred Reporting Items for Systematic Reviews and Meta-analysis (PRISMA) Guideline for evaluation of the effect zinc supplementation on COVID-19 mortality. Based on the heterogeneity a fixed-effect or random-effect model, the OR and 95% CI were used to assess the combined risk.

**Results:**

After assessment, five studies with 1506 participants in case and control groups were included in meta-analysis. The OR for one study was not estimable, and the pool OR was estimated for other studies with 1398 participants. The meta-analysis showed that zinc supplementation in cases led to a significant lower risk of mortality when it was compared with the control group; pooled OR (95% CI) was 0.57 [0.43, 0.77] (*P* < 0.001).

**Conclusion:**

This meta-analysis has suggested that zinc supplementation is associated with a lower mortality rate in COVID-19 patients. Zinc supplementation could be considered as a simple way and cost benefit approach for reduction of mortality in COVID-19 patients.

## Introduction

A member of Coronaviridae family, severe acute respiratory syndrome coronavirus 2 (SARS-CoV-2) is the agent of a pneumonia outbreak and was called 2019 novel coronavirus disease (COVID-19) [1]. It spreads mainly with respiratory droplets. COVID-19 emerged in December 2019 and considered a pandemic [1]. More than 243 million infected cases are confirmed worldwide, including 4.94 million deaths (as of October 24, 2021).

There is previous knowledge about the role of zinc in the immune system modulation. Zinc deficiency decreases CD8 + T cell responses and activation of helper T cells [2].

Zinc is one of the important components of thymulin hormone. Thymulin hormone has involved in T-cell differentiation, maturation and natural killer cell (NK cell) actions [3]. The other important role of zinc is its role in production of IFN-γ, IL-2 and also, production of IL-12 with macrophage stimulation. IL-12 induces activation of the T cytotoxic cells and NK cells. These have important roles in destruction of pathogens. Zinc deficiency leads to dysregulation of IL-10 production (an anti-inflammatory cytokine) that affects the Th1 response and macrophages functions [4]. Furthermore, zinc ions inhibit coronavirus RNA polymerase activity and in the cell culture zinc ionophores could block the replication of this virus [5].

Based on the provided evidences and the fact that zinc supplementation can reduce mortality in patients with severe pneumonia [6], it is interesting to evaluate the effect of zinc supplementation on COVID-19 mortality. There are previous studies that have evaluated this effect, but there is controversy among clinicians about the zinc supplementation in these patients. Therefore, the present study aimed at meta-analysis of the results of related studies and evaluate the pooled OR of zinc supplementation and COVID-19 mortality.

## Materials and methods

### Search strategy

This meta-analysis followed Preferred Reporting Items for Systematic Reviews and Meta-analysis (PRISMA) Guideline for evaluation of the effect zinc supplementation on COVID-19 mortality [7].

### PICOS

Population: COVID-19 patients.

Intervention: to evaluate the effect of zinc supplementation on COVID-19 mortality.

Comparators: effect of the zinc supplementation compared with standard care in COVID-19 patients.

Outcomes: COVID-19 mortality.

Study designs: a meta-analysis.

A systematic search was conducted for manuscripts through PUBMED/Medline and Google Scholar (Cochrane guideline has considered it as the gray literature) up to September 2021.

### Screening process and data extraction

Search terms included SARS-CoV-2, COVID-19, zinc and mortality. Furthermore, for manuscripts that were not found in the mentioned databases, recognition has done from review studies and also reference lists of included studies. Conference proceedings, preprints and abstracts were excluded. The piloted forms were used for data extraction.

In this study, manuscripts were considered for meta-analysis if OR (95% CI) for association of zinc supplementation and COVID-19 mortality could be obtained.

### Quality assessment

Two tools were considered for the evaluation of studies risk of bias: Cochrane collaboration risk of bias tool [8] and the Newcastle–Ottawa scale [9]. Furthermore, RevMan 5.4 was used to evaluate the risk of bias of the eligible studies [10]. The Cochrane collaboration risk of the bias tool considers these items for assessment: (a) for selection bias—random sequences generation; (b) for selection bias—allocation concealment; (c) for performance bias—blinding of participants and personnel; (d) for detection bias—blinding of outcome assessment; (e) for attrition bias—incomplete outcome data; (f) for reporting bias—selective reporting and other bias.

### Statistical analysis

In this study, *P* < 0.05 was considered as statistically significant and 95% confidence interval (95% CI) was regarded as effective size in the analysis. For assessing heterogeneity, *I*^2^ and Chi-square tests were done. *I*^2^ was categorized as low (0–50%), moderate (51–75%) or high (> 75%) for assess heterogeneity. Funnel plots and Egger regression asymmetry analysis were used for evaluation of publication bias if it was doable [11]. In this study, Stata 14.0 (StataCorp, College Station, TX, USA) and RevMan 5.4 was used.

## Results

### General characteristics of studies

**Fig. 1:**
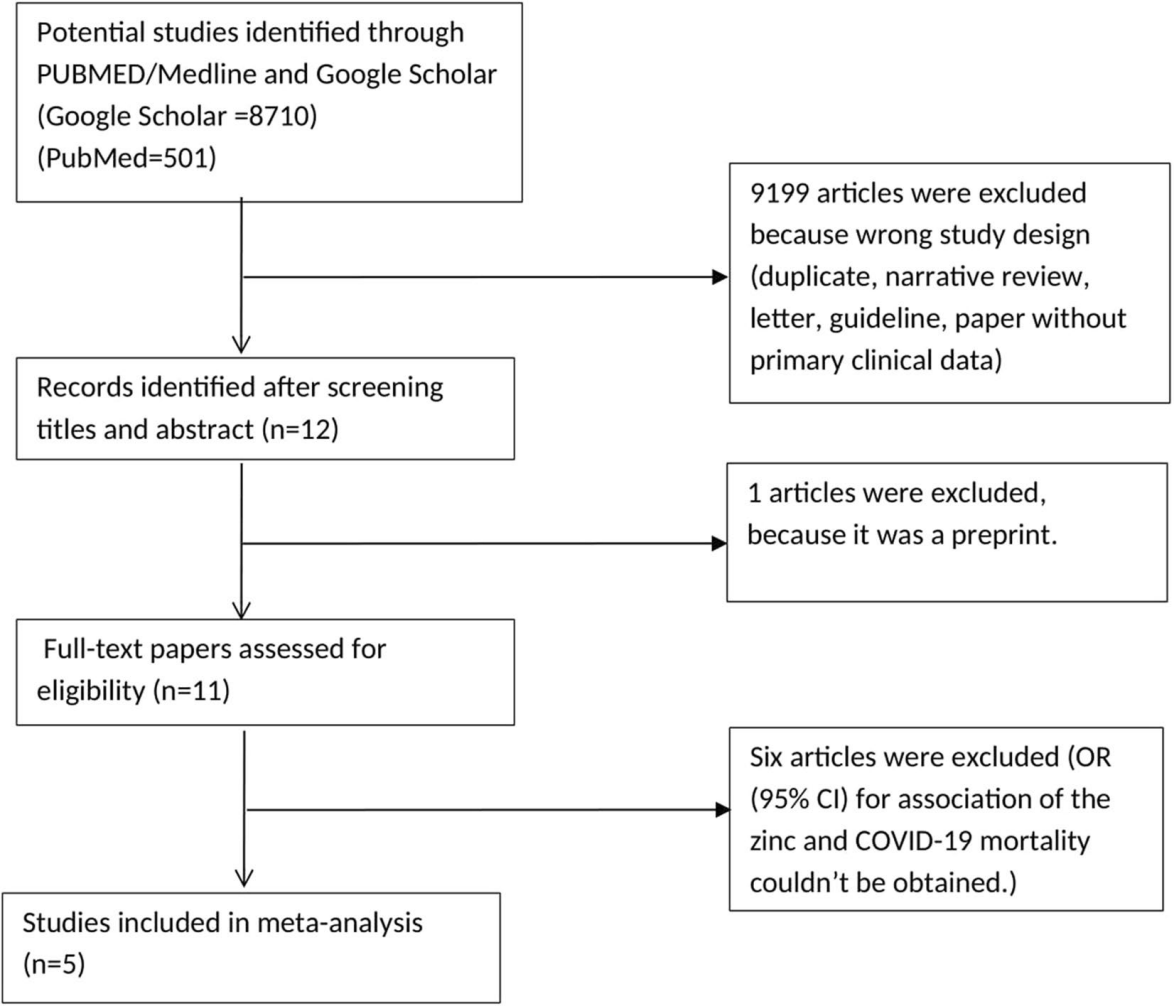
Flowchart of literature search for meta-analysis

The general characteristics (including participant’s age, studies design and sample size) of the included studies are shown in Table 1. After assessment, 5 studies with a total of 1506 participants in case and control groups were included in analysis [12–16]. The OR for one study that was conducted by Thomas et al. [15] was not estimable and the pool OR was estimated for other studies with a total of 1398 participants. The flowchart for selection of studies is shown in Fig. 1.

**Table 1:** The general characteristics of the included studies

Study	Country	Number of participants (case/control)	Study design	Age in years, mean (case/control)
Yao [16]	USA	196/46	Observational study	65/71
Carlucci et al. [13]	USA	411/521	Retrospective observational study	63.19/61.83
Abd-Elsalam et al. [12]	Egypt	96/95	Randomized controlled study	43.48/43.64
Thomas et al. [15]	USA	58/50	Randomized clinical open-label trial	44.1/42
Patel et al. [14)	Australia	15/18	Randomized controlled trial	59.8/63.8

### Meta-analysis

**Fig. 2:**
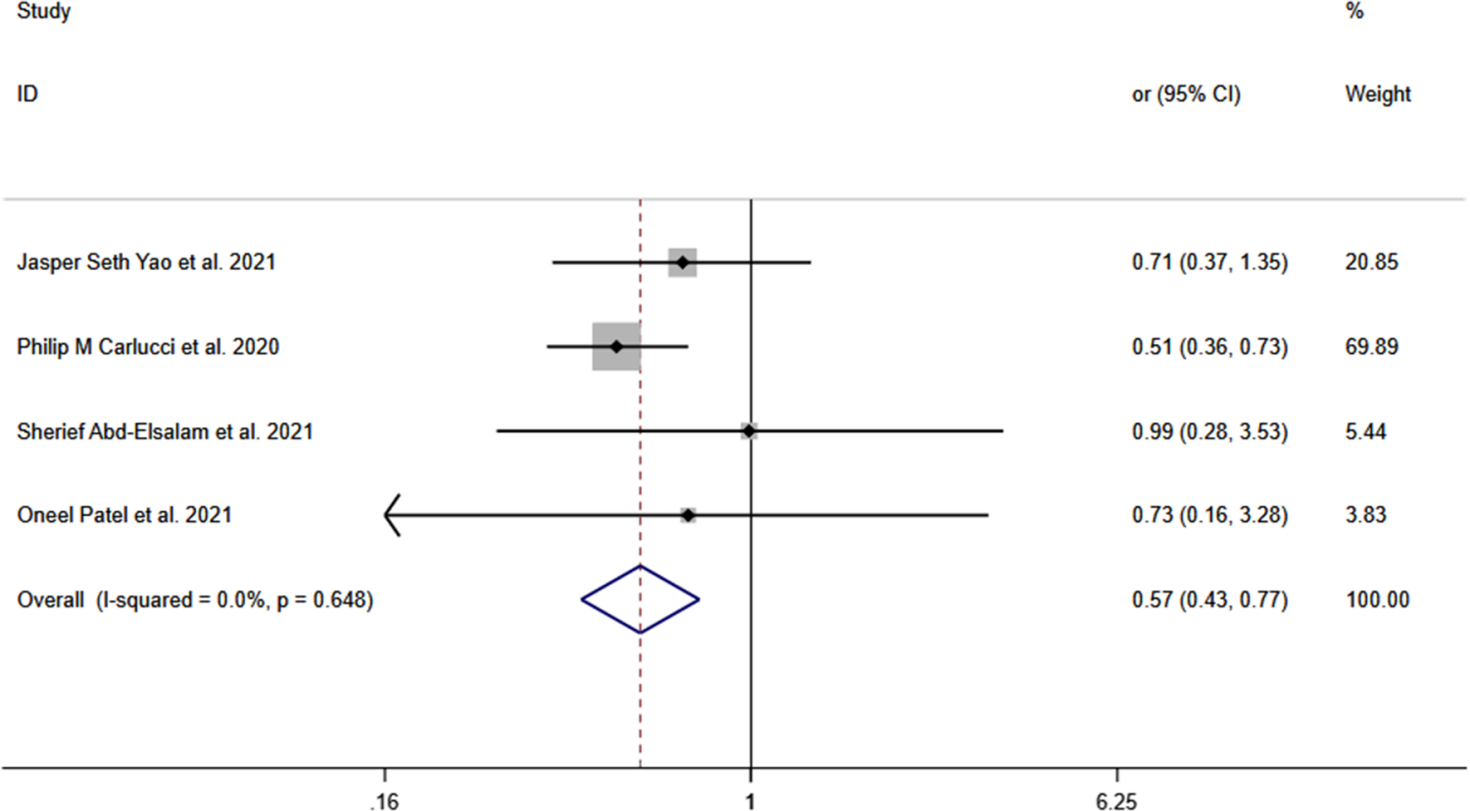
Forest plot showing the effect of zinc on COVID-19 mortality. The OR for one study that was conducted by Thomas et al. [15] was not estimable. *OR*  odds ratio

The ORs and pooled OR from the four studies are presented in Fig. 2. The OR for one study that was conducted by Thomas et al. [15] was not estimable. The meta-analysis showed that zinc supplementation in cases led to a significantly lower risk of mortality when it was compared with control group; pooled OR (95% CI) was 0.57 [0.43, 0.77] (*P* < 0.001) based on fixed-effect model. The *I*^2^ = 0.0% and *P* = 0.648 for meta-analysis indicated evidence of minimal heterogeneity.

**Fig. 3:**
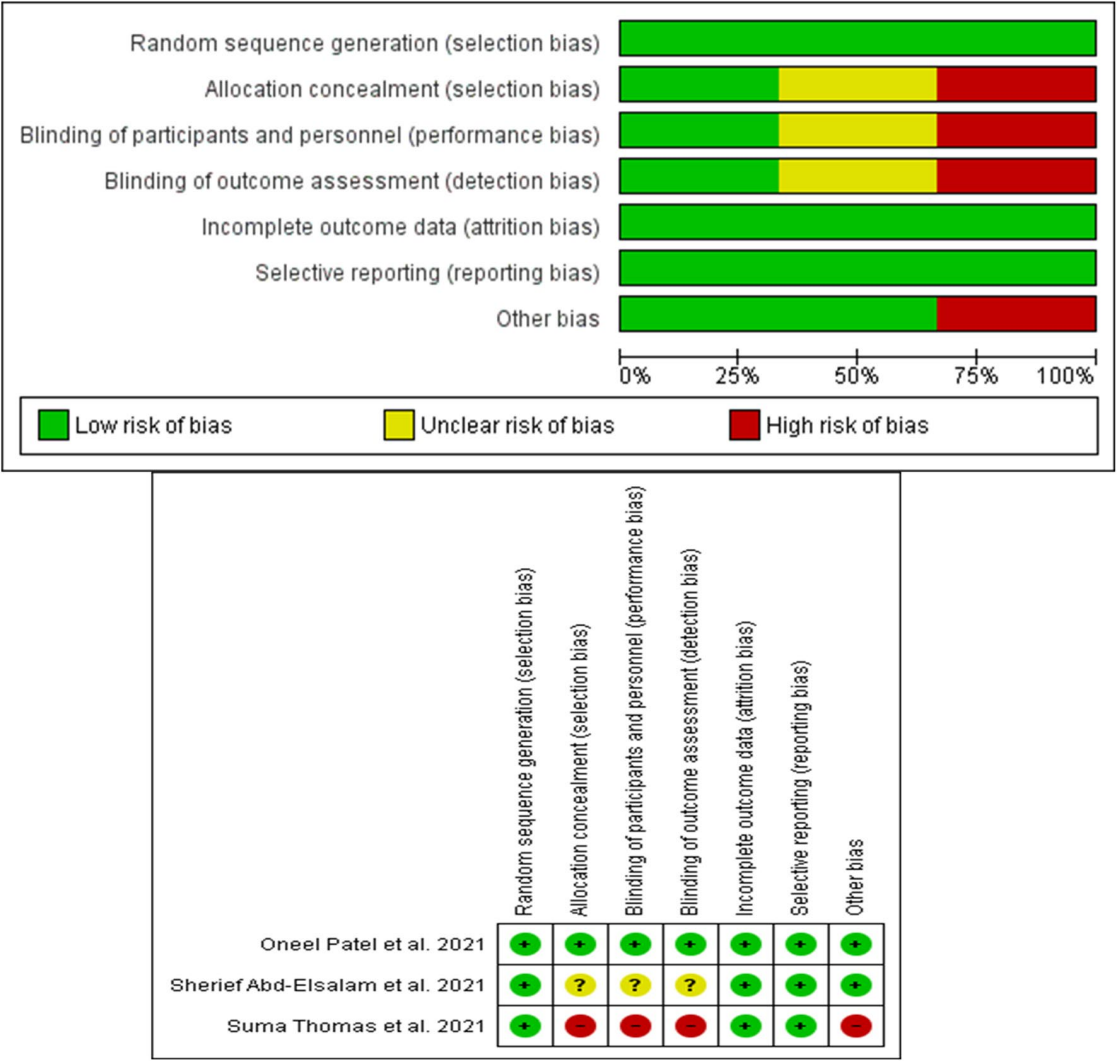
Risk of bias graph and summary: review authors’ judgements about each risk of bias item presented as percentages across eligible studies. Studies that have done by Yao et al. [16] and Carlucci et al. [13] have categorized as good studies about the risk of bias based on the modified Newcastle-Ottawa Scale by the authors’ judgement

The bias risk assessment (risk of bias graph and summary) of the eligible studies by the authors’ judgement is shown in Fig. 3. Furthermore, studies that have done by Yao et al. [16] and Carlucci et al. [13] have categorized as good studies about the risk of bias based on the modified Newcastle–Ottawa Scale by the authors’ judgement [17].

**Fig. 4:**
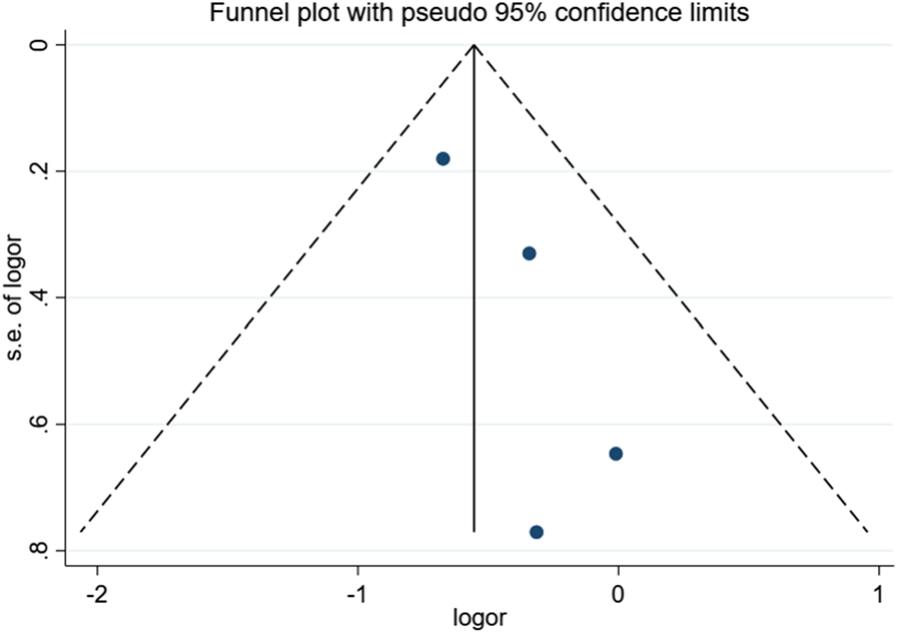
Funnel plot for publication bias

The funnel plot (visual analysis) of the included studies is shown in Fig. 4. Based on Egger’s test, publication bias was not indicated in the included studies (*P* = 0.13 in Egger’s test) [11].

## Discussion

Meta-analysis of the eligible studies has suggested that zinc supplementation is associated with a lower mortality rate in COVID-19 patients. Included studies in this meta-analysis were observational studies and randomized controlled trials. In this meta-analysis based on *I*^2^ and Chi-square tests, there was evidence of minimal heterogeneity. The result of this meta-analysis can be considered important, because it is a simple way and cost benefit approach for reduction of mortality in COVID-19 patients. There are evidences that support this result. After iron, zinc is the most abundant trace element in the human body and it has an important role in immune system modulation like CD8 + T cell responses and activation of helper T cells [2].

Its role in production of IFN-γ, IL-2, IL-12, activation of the T cytotoxic cells and NK cells could be related to destruction of pathogens. Zinc deficiency has impacts on IL-10 production that affects the Th1 response and macrophages functions [4]. Te Velthuis et al. have shown that zinc ions could inhibit the coronavirus RNA polymerase activity and also, zinc ionophores could block the replication of this virus [5]. Based on these evidences, zinc could be regarded to have an important capacity in antiviral immunity. Furthermore, zinc supplementation can decrease IL-6 and IL-1 (inflammatory cytokines) and increase type I interferon response. It could be considered as a protective mechanism in COVID-19 patients [18]. Zinc supplementation has been proposed as a preventive approach for infections because its inadequacy and deficiency affect 30% of people worldwide [19].

In a recent systematic review and meta-analysis of randomized controlled trials that has done by Hunter et al. they have assessed the benefit of zinc on the course of the acute viral respiratory tract infections [20]. They have assessed 28 RCTs, however the studies were not specific for SARS-CoV-2 infection. In their study, they have concluded that zinc may have a role in the prevention and shortening the course of viral respiratory tract infections. However, they suggest specific studies for evaluation of the effect of zinc on the SARS-CoV-2 infection. The results of the present study can confirm their results for the SARS-CoV-2 infection as the zinc supplementation is associated with a lower mortality rate in COVID-19 patients.

The result of this meta-analysis can be explained by the above-mentioned evidences that have shown antiviral activity of zinc. One of the important limitations of this study is the limited number of trials that have assessed the effects of zinc supplementation on COVID-19 patients. It can affect the reliability of conclusion in this paper. Because of few numbers of RCTs, observational studies have included in the meta-analysis. However, based on a previously published paper including both RCTs and observational studies in meta-analysis could be considered an advantage in situations like this pandemic [21]. Furthermore, The OR for one study that was conducted by Thomas et al. [15] was not estimable. There are some strengths in this meta-analysis. Evidence of minimal heterogeneity, good quality of most of the included studies and minimal risk of bias based on the Egger’s test led to a more reliable interpretation of result. More RCTs with diverse and large participants are needed for a better understanding of the effects of zinc supplementation on COVID-19 mortality and the other clinical aspects of this infection.

## Conclusions

This meta-analysis has suggested that zinc supplementation is associated with a lower mortality rate in COVID-19 patients. Zinc supplementation could be considered as a simple way and cost benefit approach for reduction of mortality in COVID-19 patients. More RCTs with large participants are needed for confirmation of this result.

## Data Availability

Not available.
